# Functional characterization of a novel somatic oncogenic mutation of *PIK3CB*

**DOI:** 10.1038/sigtrans.2017.63

**Published:** 2017-12-22

**Authors:** Andrew D Whale, Lucy Colman, Letitia Lensun, Helen L Rogers, Stephen J Shuttleworth

**Affiliations:** 1 Karus Therapeutics Ltd., Genesis Building, Library Avenue, Harwell Campus, Oxfordshire, UK

## Abstract

Class I phosphoinositide 3-kinase (PI3K) enzymes have attracted considerable attention as drug targets in cancer therapy over the last 20 years. The signaling pathway triggered by class I PI3Ks is dysregulated in a range of tumor types, impacting cell proliferation, survival and apoptosis. Frequent oncogenic mutations of *PIK3CA* have previously been discovered. In contrast, reports of *PIK3CB* mutations have been limited; however, in most cases, those that have been identified have been shown to be activating and oncogenic. The functional characterization of a *PIK3CB* catalytic domain mutant, p110β^E1051K^, first discovered by others in castrate-resistant prostate cancer (mCRPC), is outlined in this report; our data suggest that p110β^E1051K^ is a gain-of-function mutation, driving PI3K signaling, tumorigenic cell growth and migration. Tumor cells expressing p110β^E1051K^ are sensitive to p110β inhibition; its characterization as an oncogenic driver adds to the rationale for targeting p110β and indicates a continuing need to further develop specific PI3K inhibitors for clinical development in cancer therapy.

## Introduction

Class I phosphoinositide 3-kinases (PI3Ks) constitute a family of lipid kinase enzymes comprising a regulatory subunit and one of four different catalytic subunits—p110α, p110β, p110δ and p110γ—all of which are encoded by separate genes. These kinases catalyze adenosine triphosphate (ATP)-dependent phosphorylation of the 3′-hydroxyl group of membrane inositol lipids, resulting in membrane recruitment and activation of a number of lipid binding kinases, including the downstream effector kinase AKT.^1^ PI3K signaling regulates a range of important cellular processes, including transcription, translation (for example, by regulating S6 ribosomal protein), cell survival and migration *via* AKT-dependent signal transduction pathways.^2^ More recently, PI3K activation has also been reported to trigger phosphorylation of the non-receptor tyrosine kinase BMX,^3,4^ which has been implicated in cancer.^5^ The activity of PI3K in cells is antagonized by the tumor suppressor phosphatase and tensin homologue (deleted on chromosome 10) PTEN.^6^

The promise of class I PI3Ks as a molecular target family for cancer therapy has led to the design and development of a number of small molecule drugs that have advanced into clinical development.^7^ At the time of writing, the p110δ-targeted inhibitor idelalisib (Zydelig) is the sole approved PI3K inhibitor, which is specifically used for the treatment of hematological malignancies, whereas the majority of PI3K-targeted agents currently in clinical development for solid tumor therapy are pan-p110 isoform inhibitors. However, of late, more selective PI3K inhibitors have been developed; this shift has occurred in response to the emerging role of class I isoforms in both tumor cell signaling and the tumor microenvironment, and a particular focus of our research in both contexts has been p110β.

When overexpressed, wild-type p110β has been reported to be oncogenic,^8,9^ and cell surface receptor signaling to this isoform in tumor metastasis has been disclosed.^10^ Activating somatic mutations in *PIK3CA*—the gene that encodes the p110α catalytic subunit—have been reported in a number of tumors^11–13^ and are known to contribute to dysregulated tumor cell proliferation and migration.^14,15^ Historically, unlike *PIK3CA*, *PIK3CB* (the gene encoding p110β) was considered responsible for driving cancer cell proliferation and tumourigenesis in the absence of mutation, specifically in the context of tumors that express wild-type *PIK3CA* and have lost functional PTEN.^16,17^ These observations are supported by data illustrating that *PIK3CA* and *PTEN*, but not *PIK3CB*, are frequently mutated in cancer. Consistently, anchorage-independent growth assays that model tumourigenesis have shown that overexpression of mutant—but not wild-type—p110α transforms chicken embryo fibroblasts, whereas overexpression of wild-type p110β is sufficient to confer anchorage-independent growth.^9^

More recently, genome-wide screens for somatic mutations^18^ and analysis of frequently mutated residues in tumor samples^19^ have led to the identification of mutations in *PIK3CB* in tumor cells from a diverse range of cell lineages. Characterization of two resultant helical and kinase domain mutant p110β proteins, E633K and D1067V, respectively, has revealed that these *PIK3CB* mutations activate PI3K-dependent signaling, increase cancer cell proliferation and promote tumourigenic growth.^20,21^ In a multicenter genome-wide sequencing study of metastatic castrate-resistant prostate cancer (mCRPC), Robinson *et al.*^22^ identified a small patient cohort with somatic mutations in *PIK3CB*, including two patients with mutations that result in an E1051K point mutation in the kinase domain of p110β. In this study, we report the functional characterization of p110β^E1051K^. Our data suggest that p110β^E1051K^ is a gain-of-function mutation that drives PI3K signaling, tumourigenic cell growth and cell migration; moreover, cancer cells that express p110β^E1051K^ are dependent on PI3K-p110β.

## Materials and methods

### Cell culture, antibodies and reagents

Cell lines were purchased from ATCC and cultured in either RPMI-8226 or DMEM media supplemented with 10% serum, L-glutamine, penicillin and streptomycin. Sequencing of *PIK3CB* (exon 23 region) LN-18 and LoVo gDNA was performed at Eurofins Genomics (Ebersberg, Germany). For immunoblot analysis, cells were lysed in RIPA buffer (Thermo Fisher Scientific, Waltham, MA, USA) containing protease and phosphatase inhibitors (Sigma-Aldrich, St Louis, MO, USA), separated using NuPAGE Bis-Tris gels (Thermo Fisher Scientific) and transferred onto nitrocellulose membranes. Unless indicated, primary antibodies were used at a dilution of 1:1000 for western blotting. Rabbit anti-p110α, -p110β, -PTEN, -phospho-AKT^S473^, -phospho-PRAS40^T246^, and -phospho-S6^S235/S236^ and mouse anti-AKT and anti-S6(RP) were purchased from Cell Signaling Technology (Danvers, MA). Anti-GAPDH was purchased from Millipore and used at a dilution of 1:10000, and 800CW- and 680LT-conjugated secondary antibodies were purchased from Li-Cor (Lincoln, NE, USA) and diluted 1:10000. Bound fluorescent secondary antibodies were detected and quantified on western blots using the Li-Cor Odyssey SA platform and Image Studio software (Li-Cor). TGX-221 and NVP-BYL-719 were purchased from Stratech (Newmarket, UK) and Selleckchem (Houston, TX, USA), respectively, and dissolved in 100% dimethyl sulfoxide (DMSO). For cell-based assays, DMSO stock solutions were serially diluted in cell culture media containing DMSO to maintain a consistent concentration of DMSO; cells were exposed to compounds at a final concentration of 0.1% (soft agar and PC-3 and LN-18 phospho-protein ELISA), 0.2% (proliferation) or 0.3% DMSO (NCI-H460 p-AKT ELISA).

### Cloning and mutagenesis

Wild-type and E1051K-encoding *PIK3CB* cDNA was subcloned into a lentiviral transfer plasmid, p443MYCIP, and Rat2 cell lines were generated by lentiviral transduction and puromycin selection using standard techniques performed at ProQinase GmbH.

### Lipid kinase assays

Lipid kinase activity of commercially available purified PI3K-p110β and PI3K-p110β^E1051K^ complexes was determined using an ADP-Glo (Promega, Madison, WI, USA) assay performed at ProQinase GmbH. Briefly, PI3K complexes (GST-His_6_-p110β+myc-p85α) were co-expressed from *PIK3CB* and *PIK3R1* baculoviral vectors in Sf9 cells and purified by GST-affinity chromatography using standard techniques. Proteins were mixed with ATP in an assay buffer (50 mM HEPES-NaOH, pH 7.5, 1 mM EGTA, 100 mM NaCl, 3 mM MgCl_2_, 0.03% CHAPS, 2 mM DTT and PIP2:PS substrate). To determine *K*_m_ and *V*_max_, the ATP concentration was incrementally increased. For enzyme inhibition assays, compounds were added at a range of concentrations in 10% DMSO in the presence of [ATP] at the predetermined enzyme *K*_m_ and 100 μM substrate. Lipid kinase reactions were incubated at 30 °C for 40 min prior to the addition of ADP-Glo reagent and incubated for another 40 min at room temperature. Kinase detection reagent was then added and incubated for another 60 min at room temperature. Luminescence proportional to the amount of ADP generated in the reaction was quantified using a Victor2 microplate reader (Perkin Elmer, Boston, MA, USA).

### Rat2 cell line construction and anchorage-independent growth assays

Rat2 cells were transduced with lentivirus (produced using standard viral packaging vectors and protocols at ProQinase) prior to selection with 3 μg ml^−1^ puromycin 3 days post transduction. Anchorage-independent growth assays were performed by embedding cells in 0.4% agar/cell culture media. Cells were incubated for 14 days. Cell viability was determined by Alamar blue assay. Statistical significance was determined by two-tailed unpaired t test using GraphPad Prism v7 software (San Diego, CA, USA).

### Electrochemiluminescent ELISA

Whole cell lysates were analyzed for the levels of phosphorylated and total forms of AKT or S6 using multiplex Meso Scale Discovery assays and a Sector Imager 2400 plate reader in accordance with the manufacturer’s instructions (MSD, Rockville, MD, USA).

### Growth inhibition and migration assays

Proliferation of cancer cell lines seeded onto white walled view plates (Perkin Elmer, Waltham, MA, USA) was monitored in the presence of DMSO or compounds dissolved in DMSO and diluted in cell culture media to yield a final DMSO concentration of 0.2%. Widefield images were acquired every 2 h, and the percentage confluence was quantified using the IncuCyte ZOOM platform equipped with a ×10 objective (Essen Biosciences, Ann Arbor, MI, USA). When relevant, cell viability was determined after 72- h exposure to compounds with Cell Titer Glo assay (Promega). Luminescent data were then expressed as a percentage of DMSO control and analyzed using GraphPad Prism v7 software and non-linear fit to generate an IC_50_. Where indicated, statistical significance was determined by one-way analysis of variance (ANOVA) and Tukey’s multiple comparison test using GraphPad Prism v7 software.

Migration assays were performed using the IncuCyte ZOOM. Cells were seeded onto 96-well ImageLock plates, and a scratch was introduced into each well using a Woundmaker tool (Essen Biosciences). Cells were exposed to compounds with an image taken every 2 h for 72 h. Cell migration into the wound was quantified using the IncuCyte ZOOM cell migration software module and the % relative wound density metric (Essen Biosciences).

## Results

### Identification of a *PIK3CB* mutation in patient samples that encodes a point mutation in the catalytic domain of p110β

*PIK3CB* encodes the p110β lipid kinase catalytic subunit of PI3Kβ. Mutation of *PIK3CA*—the gene that encodes PI3K subunit p110α—drives cancer cell proliferation,^23^ whereas several reports support the hypothesis that *PIK3CB* is responsible for driving tumourigenesis in the absence of mutations and specifically in the context of tumors that contain wild-type *PIK3CA* and have lost functional PTEN.^16,17^ However, more recently, somatic mutations in *PIK3CB* have been identified in patients with cancers from a diverse range of lineages, including prostate, esophageal and renal carcinoma.^18,22,24^

**Figure 1:**
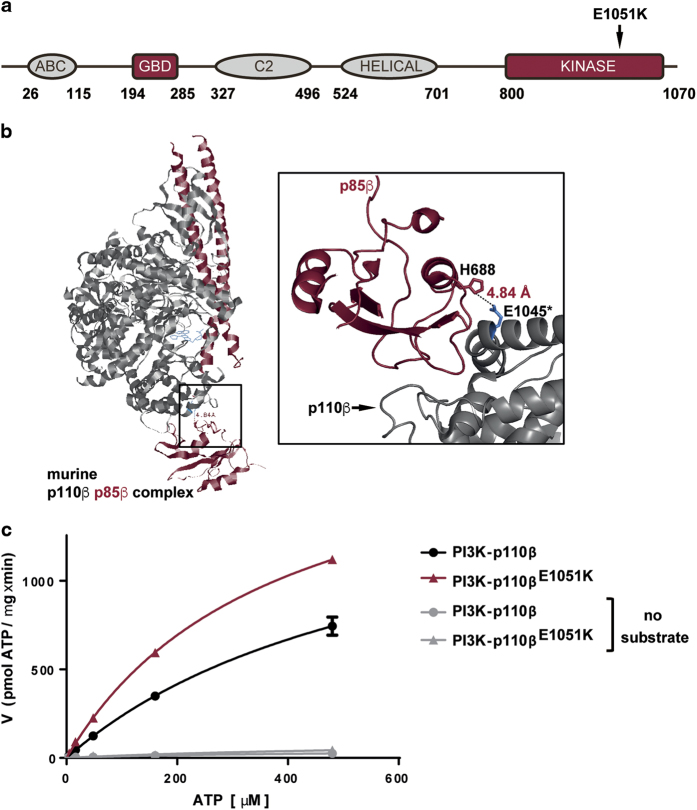
Somatic mutation in the *PIK3CB* coding DNA sequence (CDS) at position 3151 (G to A substitution) results in an activating E1051K point mutation in p110β. (**a**) Diagram illustrating the domain structure of p110β and location of the E1051K mutation. (**b**) Ribbon diagram of the kinase domain structure of murine p110β (gray) in complex with murine p85β (red) bound to the ATP competitive inhibitor GDC-0941 (2Y3A). Close-up region depicts the location of the p110β E1045 acidic side chain (*murine equivalent of E1051K) and the proximal p85 His basic side chain. (**c**) Lipid kinase activity of wild-type p110β and p110β^E1051K^ in the presence or absence of saturating PIP2:PS substrate and varied concentrations of ATP (shown is a representative example of two independent experiments performed in duplicate). Error bars indicate s.d. of duplicate samples.

While analyzing publicly available datasets for mutations in *PIK3CB*—namely, COSMIC (cancer.sanger.ac.uk,^25^) and The Cancer Genome Atlas (TCGA, viewed *via* cbioportal.org)—we noted a somatic mutation in the coding DNA sequence (CDS) at position 3151 (G to A substitution), resulting in an E1051K point mutation in p110β, that was present in 13 of the 251 samples containing mutations in *PIK3CB* (from a total 31780 samples tested). At the equivalent position in *PIK3CA*, G1049 was mutated to arginine in 84 of 105085 tested samples. This *PIK3CB* mutation was originally identified in two patients with mCRPC^22^ and occurred at a similar frequency to a reported oncogenic mutation of D1067 in the COSMIC database (10 samples contain D1067V/Y/A/H mutation in *PIK3CB*). E1051K mutations were also present in tumors from a diverse range of lineages and in cell lines derived from patients with glioblastoma and colon adenocarcinoma (Table 1). E1051 is located in a helix within the kinase domain of p110β, which is proximal to the region bound by the p85 subunit in the murine crystal structure,^26^ suggesting that mutation of this residue might impact kinase activity (Figure 1a and b). To determine the effect of the E1051K mutation on p110β lipid kinase activity, we expressed p110β from vectors encoding wild-type *PIK3CB* or *PIK3CB* in which a 3151G>A mutation was introduced, and co-purified p110β with p85α. Enzyme *K*_m_ [ATP] and *V*_max_ for p110β and p110β^E1051K^ were determined in the presence of substrate and increasing concentrations of ATP using a homogenous luminescence assay to monitor the formation of ADP product. Repeat assays from multiple independent test occasions and protein preparations consistently revealed that purified recombinant p110β^E1051K^ had a higher *V*_max_ and lower *K*_m_ [ATP] in comparison to wild-type p110β (Figure 1c and Table 2). Importantly, in the absence of substrate, no increase in ADP formation was detected (Figure 1c). Although it is not possible to exclude the possibility that the preparations of p110β^E1051K^ contained higher levels of correctly folded/active protein (or fewer contaminating proteins, Supplementary Figure 1), the trend in these data suggests that the p110β^E1051K^ mutation does not inhibit p110β kinase activity and that p110β^E1051K^ has a higher affinity for ATP and greater activity than wild-type p110β. Finally, we determined the ability of a selective p110β inhibitor, TGX-221,^27^ to inhibit the lipid kinase activity of p110β^E1051K^ in the presence of ATP at the enzyme *K*_m._ To compare the activity of TGX-221 on the wild-type p110β enzyme to p110β^E1051K^, *K*_i_ values were calculated from the IC_50_ values measured at K_m_ [ATP] using the Cheng-Prusoff equation. Interestingly, we found that, *in vitro*, TGX-221 is able to inhibit p110β^E1051K^ with comparable potency to wild-type p110β (Table 2).

### p110β^E1051K^ drives oncogenic anchorage-independent growth

**Figure 2:**
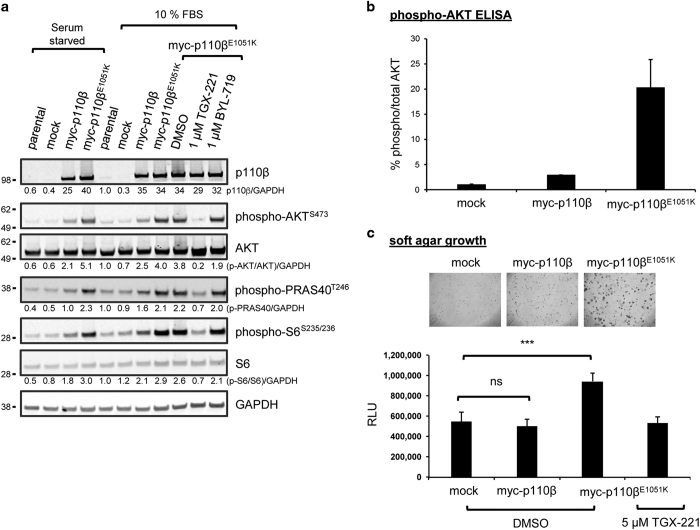
Exogenous expression of p110β^E1051K^ promotes PI3K pathway activation and anchorage-independent growth. (**a**) Rat2 cells stably expressing myc-p110β or myc-p110β^E1051K^ or cells transduced with empty vector (mock) were maintained in growth conditions (10% FBS) or serum starved in 0.5% FBS overnight prior to lysis. Lysates of cells were separated by SDS-PAGE and probed with antibodies to detect p110β expression, phosphorylated proteins, or GAPDH loading control as indicated. Fluorescent intensity of bands was quantified and normalized to GAPDH and then to signals from parental Rat2 cell lysates and is shown below blots to 2 sig. fig. (**b**) The level of AKT^S473^ phosphorylation in transduced Rat2 cell lysates was determined using a multiplexed phospho-/total electrochemiluminescent ELISA assay. Data are the mean±s.e.m. of duplicate samples from two independent experiments. (**c**) Soft agar growth assay to compare the effect of stable expression of myc-p110β and myc-p110β^E1051K^ on the growth of Rat2 cells in soft agar with control cells harboring empty vector (mock). Rat2 cells were plated in soft agar, and growth was imaged after 14 days by light microscopy and quantified with Alamar Blue viability assay. Data are the mean±s.e.m. of duplicate samples from two independent experiments. On one test occasion, Rat2 cells expressing myc-p110β^E1051K^ were also cultured in the presence of 5 μM TGX-221. ***indicates *P*=0.0005; ns, not significant.

To determine whether the p110β^E1051K^ mutation is an oncogenic driver, we stably transfected murine Rat2 fibroblast cells with a myc-tagged *PIK3CB* expression vector encoding either myc-p110β or myc-p110β^E1051K^ or an empty vector as a control (mock). Analysis of transfected cell lysates cultured either in the presence of serum or in serum-starved conditions revealed that overexpression of myc-p110β in Rat2 cells to a level approximately 35-fold higher than that of the endogenous protein in Rat2 cells and a human fibrosarcoma cell line (Supplementary Figure 2) resulted in increased endogenous phosphorylation of AKT (p-AKT), proline-rich AKT1 substrate (p-PRAS40) and S6 ribosomal protein (p-S6) in the PI3K signaling pathway (Figure 2a). Moreover, the level of p-AKT^S473^ (immunoblot in Figure 2a, and ELISA in Figure 2b), p-PRAS40^T246^ and p-S6^S235/S236^ (Figure 2a) were greater in lysates from cells expressing myc-p110β^E1051K^ in comparison to those expressing myc-p110β. Importantly, the level of myc-p110β and myc-p110β^E1051K^ expression in the respective Rat2 cell lines appeared to be equivalent as determined by western blot (Figure 2a) and flow cytometry (data not shown). To confirm that the increase in AKT and S6 phosphorylation was the result of increased p110β lipid kinase activity, myc-p110β^E1051K^ expressing cells were incubated with either p110β inhibitor, TGX-221,^27^ a selective p110α inhibitor, BYL-719^28^ or dimethyl sulfoxide (DMSO) as a control. According to previous literature reports, TGX-221 inhibits purified p110β with an IC_50_ value of 5 nM and displays ~1000-fold selectivity for p110β over the p110α isoform,^27^ whereas BYL-719 inhibits purified p110α with an IC_50_ value of 5 nM and has 240-fold selectivity for p110α over the p110β isoform.^28^ In our study, western blot analysis revealed that AKT, PRAS40 and S6 phosphorylation was diminished in lysates of myc-p110β^E1051K^ expressing cells treated with TGX-221. In contrast, the level of AKT, PRAS40 and S6 phosphorylation in cells treated with BYL-719 was indistinguishable from either DMSO treated or untreated control myc-p110β^E1051K^ expressing cells (Figure 2a).

Anchorage-independent growth of cancer cells *in vitro* is an established aspect of the tumor phenotype. To determine the effect of E1051K mutation on the transforming potential of p110β, we cultured murine Rat2 fibroblast cells stably expressing either myc-p110β or myc-p110β^E1051K^ or an empty vector as a control (mock) in a soft agar colony formation assay with a fluorescent cell viability endpoint measurement. Rat2 cells expressing myc-p110β^E1051K^ produced larger colonies than cells expressing wild-type myc-p110β (Figure 2c). Consistently, quantification of cell viability revealed that Rat2 cells expressing myc-p110β^E1051K^ showed enhanced proliferation in soft agar after 14 days in comparison to cells expressing wild-type myc-p110β or mock transduced Rat2 cells (Figure 2c). These data suggest that p110β^E1051K^ drives PI3K-dependent tumourigenic cell growth.

### PI3K signaling and proliferation in cancer cells with 3151G>A mutation is dependent on p110β^E1051K^

**Figure 3:**
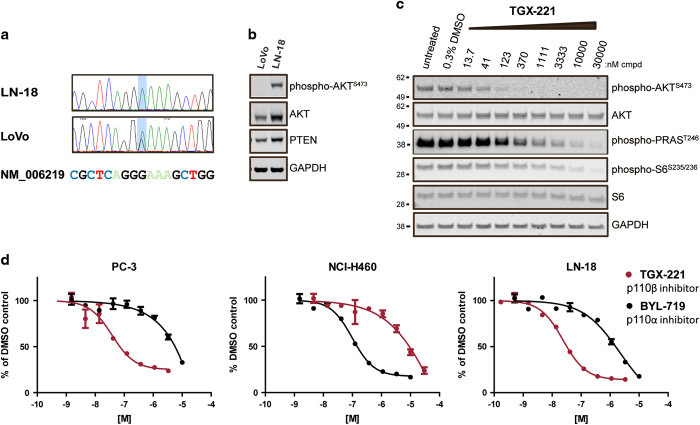
PI3K signaling in cells with an endogenous *PIK3CB* mutation is dependent on p110β. (**a**) Sequence of *PIK3CB* in LN-18 and LoVo cell gDNA identified 2 missense mutations at the same codon located within the kinase domain. Light blue shading indicates the location of the nucleotide change. (**b**) Immunoblot of lysates of LoVo and LN-18 cells cultured overnight in media containing 10% fetal bovine serum probed using antibodies to detect the level of p-AKT, AKT, PTEN and GAPDH expression. A total of 20 μg of protein was loaded in each lane. (**c**) Immunoblot of lysates of LN-18 cells treated with cell culture media containing 0.3% DMSO or TGX-221 at indicated concentration in 0.3% DMSO were probed using antibodies to detect inhibition of p-AKT, p-PRAS40 and p-S6. (**d**) p-AKT ELISA in PC-3 (PTEN null, p110α^wt^, and p110β^wt^), LN-18 (PTEN^wt^, p110α^wt^, and p110β^E1051K^) and NCI-H460 cells (PTEN^wt^, p110α^E545K^, and p110β^wt^). Cells were treated with TGX-221 or BYL-719 in DMSO or DMSO alone for 2 h and lysed, and the lysates were probed with antibodies to detect AKT or phospho-AKT^S473^ using a multiplex electrochemiluminescent ELISA assay. RLU signal was quantified using an MSD SI2400. p-AKT signal was normalized to total AKT (expressed as % p-AKT) and then indexed to DMSO treated cells. Shown are dose response curves that are representative examples of replicates from individual experiments (error bars indicate standard deviation of duplicate samples; where they are not clearly visible, the error in the replicates is too low to be seen on the plot). Mean data of duplicate samples from two independent experiments are listed in Table 3.

To dissect the role of *PIK3CB* in cancer cells expressing endogenous p110β^E1051K^, we utilized two cell lines identified in the COSMIC cell lines project dataset (cancer.sanger.ac.uk,^25^ Table 1), namely, LN-18 and LoVo. We initially sought to confirm the presence of the 3151G>A mutation by sequencing the *PIK3CB* gene. Analysis of the sequence in exon 23 revealed a G to A substitution in the sequence of LN-18 compared to the reference sequence, making it a useful tool to dissect the role of p110β^E1051K^ (Figure 3a). In LoVo cells, peaks corresponding to both G and A were detected at position 3151, which is consistent with the fact that this cell line is heterozygous for this mutation (Figure 3a). We next sought to determine the impact of p110β inhibition on PI3K signaling in cell lines with a 3151G>A *PIK3CB* mutation. In preliminary experiments, we probed lysates of LN-18 and LoVo cells with antibodies to detect AKT and p-AKT^S473^ by western blot (Figure 3b) or ELISA (data not shown). The level of AKT expression was lower in lysates of LoVo cells in comparison to LN-18, and the detectable p-AKT signal in LoVo cells cultured in serum was too low to allow for effective further analysis. To determine the impact of p110β inhibition on PI3K signaling in LN-18 cells, we again utilized the p110β inhibitor TGX-221 and exposed LN-18 cells to a range of concentrations of this compound or DMSO as a control. Western blot analysis of lysates of LN-18 cells treated with TGX-221 and probed with antibodies to detect p-AKT^S473^, p-PRAS40^T246^ and p-S6^S235/S236^ revealed that TGX-221 inhibited AKT and S6 phosphorylation in a dose-dependent manner (Figure 3c).

Signaling downstream of PI3K in cells that harbor mutations in *PIK3CA* is dependent on p110α,^23^ whereas PI3K signaling in PTEN-null prostate cancer cell lines is dependent on p110β.^16,17,29^ In agreement with these data, p-AKT in PTEN-null PC-3 cells was more sensitive to inhibition with TGX-221 than BYL-719 in a multiplex ELISA assay using specific antibodies to detect phospho-S473 and total AKT. In contrast, PTEN and p110α^E545K^ expressing NCI-H460 cells were more sensitive to inhibition with BYL-719 than TGX-221 (Figure 3d, Table 3). Similar to PC-3 cells, but in contrast to NCI-H460 cells, TGX-221 inhibited p-AKT^S473^ more potently than BYL-719 in wt PTEN expressing LN-18 cells (Figure 3d, Table 3). These results indicate that PI3K signaling downstream of p110β^E1051K^ is intact in LN-18 cells and moreover is dependent on p110β.

**Figure 4:**
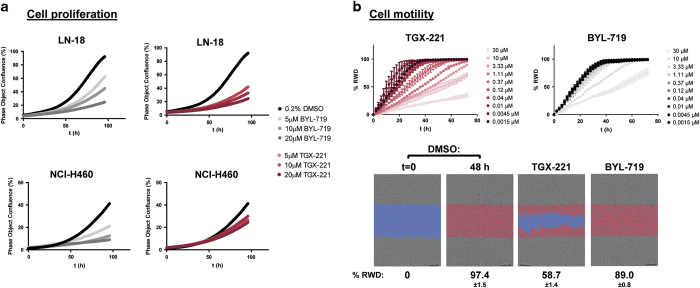
Time- and concentration-dependent inhibition of proliferation and cell migration. (**a**) LN-18 and NCI-H460 cell lines were treated with TGX-221 or BYL-719 in 0.2% DMSO or only 0.2% DMSO as a control, and the effect on cell proliferation was monitored using an IncuCyte ZOOM kinetic live cell imaging system to image and quantify confluence at 2-h intervals for a 96-h period. (**b**) Scratch wounds were created in monolayers of LN-18 cells. Cells were then incubated for a 72-h period in the presence of TGX-221 or BYL-719 in 0.3% DMSO or only 0.3% DMSO as a control, and cell migration into the scratch wound was monitored using an IncuCyte ZOOM. Images were acquired every 2 h for a 72 h period. The movement of cells was tracked using IncuCyte ZOOM software and analyzed using the percentage relative wound density (RWD) metric (line plots). Representative images of DMSO at *t*=0 and DMSO, TGX-221 and BYL-719 treated cells at 48 h are shown. The scratch is highlighted blue, and cells invading into the wound in each image are colored red. Data shown are representative of two independent experiments performed in duplicate (percentage±s.d.).

**Figure 5:**
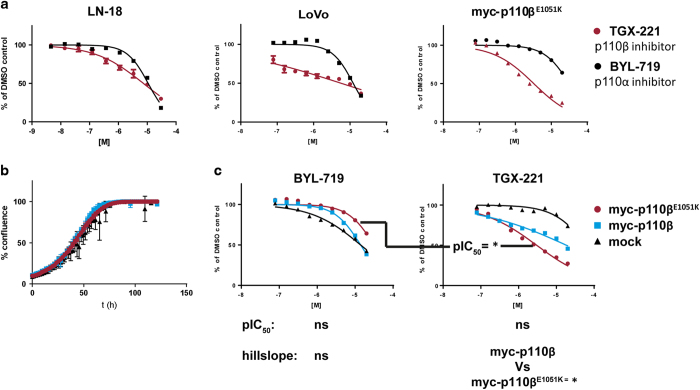
(**a**) Inhibition of proliferation of cell lines expressing p110β^E1051K^. (**a**) LN-18 and LoVo that express endogenous p110β^E1051K^ and Rat2 cells stably expressing exogenous myc-p110β^E1051K^ were treated with a range of concentrations of TGX-221 or BYL-719 in 0.2% DMSO or only 0.2% DMSO as a control and allowed to proliferate for 72 h. Cell viability was measured using a Cell Titer Glo assay. (**b**) Proliferation of mock transduced Rat2 cells or Rat2 cells stably expressing myc-p110β or myc-p110β^E1051K^ was monitored using an IncuCyte ZOOM kinetic live cell imaging system to image and quantify confluence at 2- h intervals for a period of 5 days. (**c**) Mock transduced Rat2 cells or Rat2 cells stably expressing myc-p110β or myc-p110β^E1051K^ were treated with a range of concentrations of TGX-221 or BYL-719 in 0.2% DMSO or only 0.2% DMSO as a control and allowed to proliferate for 72 h. Cell viability was measured using a Cell Titer Glo assay. Rat2 cells expressing myc-p110β^E1051K^ are more sensitive to TGX-221 than BYL-719, whereas mock transduced Rat2 cells are more sensitive to BYL-719 than TGX-221. Shown are dose–response curves that are representative examples of replicates from individual experiments (error bars indicate s.d. of replicate samples; where they are not clearly visible, the error in the replicates is too low to be seen on the plot). ANOVA (with Tukey’s multiple comparison) analysis of pIC_50_ and hillslope parameters derived from all data from two independent experiments are listed below plots. ‘*’ indicates *P*=<0.05; NS, not significant.

As PI3K-mediated AKT phosphorylation is dependent upon p110β in LN-18 cells, we sought to determine whether inhibition of p110β activity impacted cell proliferation and migration, two processes that are critical to the cancer cell phenotype. Treatment of LN-18 cells with TGX-221 inhibited both cell proliferation and migration in a time- and dose-dependent manner in widefield, image-based time lapse video microscopy assays; moreover, greater potency was observed for TGX-221 than for the p110α-specific inhibitor BYL-719 in this context (Figure 4a and b). Similarly, in proliferation assays, TGX-221 inhibited the growth of both LoVo and LN-18 cells more potently than BYL-719 (Figure 5a). Finally, we tested the ability of either TGX-221 or BYL-719 to inhibit the proliferation of Rat2 cells stably expressing either myc-p110β or myc-p110β^E1051K^ or mock transduced Rat2 cells in a 2D proliferation assay. In contrast to growth in soft agar, expression of either myc-p110β or myc-p110β^E1051K^ did not affect the rate of cell proliferation in 2D culture (Figure 5b). Comparison of the growth inhibition data revealed that mock Rat2 cells were the most sensitive to BYL-719. Importantly, and in contrast to the mock transduced cell line, the expression of myc-p110β and myc-p110β^E1051K^ sequentially decreased sensitivity to BYL-719. Concomitantly, the expression of myc-p110β and myc-p110β^E1051K^ increased sensitivity to TGX-221; proliferation of myc-p110β^E1051K^ expressing Rat2 cells was least sensitive to BYL-719 and most sensitive to TGX-221-mediated inhibition (Figure 5a and c).

Taken together, these data suggest that p110β^E1051K^ is an activating oncogenic mutation and that cancer cells expressing p110β^E1051K^ are dependent on p110β for tumourigenic growth.

## Discussion

The role of PI3K-p110β in cancer had been thought to be confined to the specific context of tumors lacking both functional PTEN and activating mutations in *PIK3CA*, the gene that encodes the p110α catalytic subunit.^16^ As an example, in PTEN-null prostate cancer cells, signaling downstream of PI3K and cell proliferation are dependent on p110β.^17,29^ More recently, several studies adopting different whole genome-wide screening strategies have identified somatic mutations in *PIK3CB*.^18,19,22^ In one study of mCRPC, two patient samples were identified that harbored a specific mutation at position 3151 in the CDS that results in point mutation E1051K in p110β.^22^

In this study, we report that the E1051K mutation has also been detected in tumors from patients with breast, lung, esophageal, renal and stomach malignancies. Using a suite of *in vitro* and cell-based assays, we characterized the function of the p110β^E1051K^ mutation. The mutation appears to increase the catalytic activity of p110β *in vitro*, up-regulate downstream PI3K signaling—as evidenced by an increase in the level of phosphorylated AKT and S6 in comparison to wild-type p110β—and promote anchorage-independent growth when expressed *de novo* in murine fibroblast cells incapable of proliferating in soft agar. Furthermore, established cell lines that harbor endogenous *PIK3CB* mutations encoding p110β^E1051K^ exhibit PI3K signaling, motility and proliferation that is sensitive to inhibition by the p110β-selective inhibitor TGX-221, but refractory to BYL-719, an inhibitor of p110α. Taken together, these data suggest that p110β^E1051K^ is an activating gain-of-function mutation that drives tumourigenic growth.

Importantly, although PI3K signaling and proliferation of LN-18 and LoVo cells –which harbor wild-type *PIK3CA*, p110β^E1051K^-encoding *PIK3CB* and express PTEN –were more sensitive to inhibition with TGX-221 than BYL-719, proliferation of NCI-H460 cells (which express wild-type p110β and wild-type PTEN and harbor the p110α^E545K^ mutation) was more sensitive to BYL-719 than TGX-221. Differential sensitivity to TGX-221 is not limited to this compound; similar data were obtained with a number of PI3K-p110β inhibitors (data not shown). Furthermore, differential selectivity is neither explained by higher target (p110β) expression levels in LN-18 cells (data not shown) nor by increased avidity of TGX-221 for the mutant enzyme over wild type, since in lipid kinase assays, TGX-221 inhibited both p110β and p110β^E1051K^ with equivalent potency (Table 2). Consistently, we also found that in a commercially available cell line panel consisting of LoVo cells and 12 cell lines that harbor a range of mutations in *PIK3CA* present in tumor cells of cancer patients, *PIK3CB*-mutated LoVo cells were the most sensitive cells in the panel to TGX-221; in contrast to the cell lines with *PIK3CA* mutations, LoVo cells were the least sensitive to BYL-719-mediated inhibition of cell proliferation (data not shown). Interestingly, in proliferation assays where cells were grown in adherent culture, the sensitivity of Rat2 cell proliferation to inhibition of p110α (by BYL-719) or inhibition of p110β (by TGX-221) was reversed by *de novo* expression of p110β^E1051K^ (Figure 5b). Taken together, these data suggest that the differences between cell lines is the result of differential dependency on the two p110 isoforms for cells that harbor mutations in *PIK3CA* (in which proliferation appears to be dependent on p110α) in comparison to cells with p110β^E1051K^-encoding *PIK3CB* mutations in which proliferation appears to be dependent on p110β.

Based on sequence alignments, p110β^E1051K^ is an analogous mutation to G1049R in p110α.^22^ G1049R mutations are observed at a notably lower frequency than ‘hotspot’ p110α E545K and H1049R mutations.^25,30^ However, functional characterization of G1049R indicates that p110α^G1049R^ is an activated oncogenic variant of p110α.^30^ Additional mutations in *PIK3CB* close to the p110β^E1051K^-encoding region have also been reported, albeit at low frequency in patient samples to date. Of note, Kim *et al.*^31^ demonstrated that p110β^A1048V^ is a transforming gain-of-function mutant; oncogenic p110β^D1067V^ was found to be responsible for resistance to EGFR inhibition in cell lines and erlotinib therapy in a patient with non-small cell lung cancer;^21^ one patient enrolled in a phase I clinical trial of the p110β inhibitor GSK2636771 achieved a durable response and had genetic lesions that resulted in p110β^L1049R^;^32,33^ finally, Nakanishi *et al.*^34^ identified a p110β^D1067Y^ mutation in cells cultured in the presence of the pan-PI3K isoform inhibitor pictilisib (GDC-0941) that conferred resistance to the anti-proliferative effects of pictilisib. The clinical significance of p110β^E1051K^—the most frequent amino-acid change resulting from *PIK3CB* mutation identified in the COSMIC dataset—and other low frequency, yet apparently oncogenic mutations in p110β, remains to be elucidated. The central role of PI3K in regulating cancer cell proliferation, metastasis and genomic instability make PI3K an important clinical target in cancer. The increasing identification and characterization of gain-of-function mutations in the C-terminal region of p110β suggest that this region of the kinase domain is critical for regulating PI3K-p110β lipid kinase activity and may become of increasing clinical importance as the development of more selective PI3K inhibitors gains pace. This study highlights the importance of the personalized medicine approach because defining the specific genetic/mutational context in the patient can have important implications for the efficacy of isoform-selective PI3K inhibitors.

**Note added to proof**

While this paper was under review, the FDA approved the PI3K pan-inhibitor copanlisib for the treatment of hematological malignancies.

## Figures and Tables

**Table 1:** List of tumor samples and cell lines with 3151G>A mutation of *PIK3CB*^a^

*Sample ID*	*Cancer lineage*^b^	*AA alteration*
TCGA-D8-AIJC-01	Breast	E1051K
BK00300	Kidney	E1051K
S00356 (COSS1759184)	Lung	E1051K
S00356 (COSS2480812)	Soft tissue	E1051K
LP6007420-DNA-A01	Esophageal	E1051K
ESCC123	Esophageal	E1051K
SC9063	Prostate	E1051K
SC9071	Prostate	E1051K
TCGA-BR-8364-01	Stomach	E1051K
TCGA-BT-A0YX-01	Urinary tract	E1051K
TCGA-CJ-4900-01	Colon	E1051G
LN-18	Glioblastoma	E1051K
LoVo	Colon	E1051K

a source: COSMIC (Forbes *et al.*, 2015); cbioportal.org was used to view TCGA datasets.

b Carcinoma, unless indicated.

**Table 2:** Activity and inhibition of p110β enzymes

*Enzyme*	K_*m*_^a^ *[ATP] (μM)*	V_*max*_^a^ *(pmol μg*^*−1*^* min)*	*TGX-221*^b^	
			*IC*_*50*_ *(nM)*	*K*_*i*_ *(nM)*
p110β	507.5±66.3	1134±338	24.15±2.9	12.27±1.47
p110β^E1051K^	314.3±36.8	1401.8±335	19.54±7.24	9.32±3.45

a Purified p85α-p110β complexes containing either wild-type p110β or p110β^E1051K^ were incubated with increasing amounts of ATP. ATP consumption in the presence of PIP2:PS substrate was detected by measurement of ADP generation. Data are mean from four independent experiments performed in duplicate±s.e.m.

b Inhibition of lipid kinase activity was determined using a homogeneous luminescence assay to monitor the formation of ADP in the presence of ATP at the enzyme *K*_m._ *K*_i_ values were calculated from the IC_50_ values measured at *K*_m_ [ATP] using the Cheng-Prusoff equation. Data are mean from two independent experiments performed in duplicate±s.e.m.

**Table 3:** Inhibition of AKT phosphorylation in cell-based assays

*Cell line*	*IC*_*50*_*(nM)*^a^											
	*p-AKT*^*T308*^						*p-AKT*^*S473*^					
	*TGX-221*			*BYL-719*			*TGX-221*			*BYL-719*		
	n*=1*	n*=2*	*mean*	n*=1*	n*=2*	*mean*	n*=1*	n*=2*	*mean*	n*=1*	n*=2*	*mean*
PC-3	−	−	−	−	−	−	15.55	37.9	**26.7**	>10000^b^	>10000^b^	**>10000**^b^
LN-18	20.88	11.44	**16.2**	950.7	3792	**2374.4**	23.93	32.58	**28.3**	2165	1647	**1906**
NCI-H460	—	—	—	—	—	—	>30000^b^	>30000^b^	**>30000**^b^	100.3	118.7	**109.5**

—, Not done.

a Levels of phospho-AKT^T308 and S473^ were measured in cell lysates treated with a range of concentrations of the indicated compound in 0.1% DMSO (PC-3 & LN-18 cells) or 0.3% DMSO (NCI-H460) normalized to the level of total AKT and expressed as a percentage of DMSO control. IC_50_ values were generated from independent experiments (*n*) performed in duplicate by non-linear curve fit using Prism software (GraphPad, SD).

b Top concentration tested in assay.
