# Supplementary figures and images for: Functional characterization of a novel somatic oncogenic mutation of PIK3CB
Source: Signal Transduct Target Ther. 2017 Dec 22;2:17063–. doi: 10.1038/sigtrans.2017.63 (PMC5740215; doi:10.1038/sigtrans.2017.63)

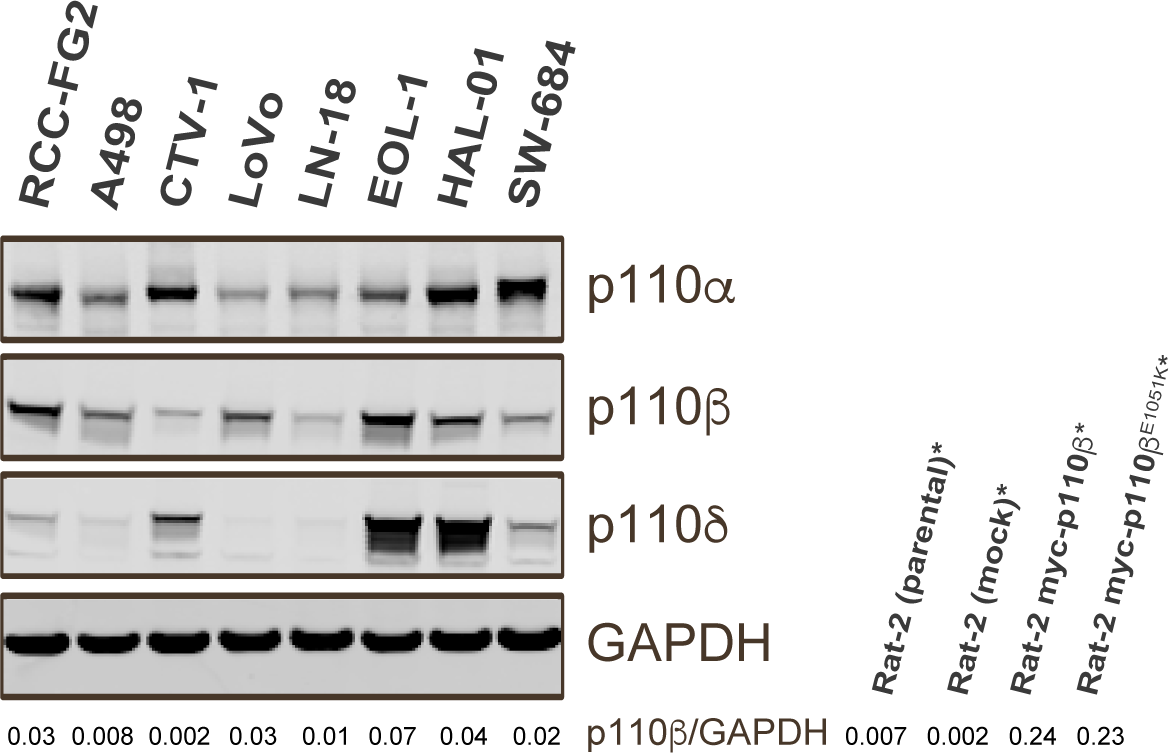

Supplement: Supplementary Figure 1 [file sigtrans201763-s1.tiff]

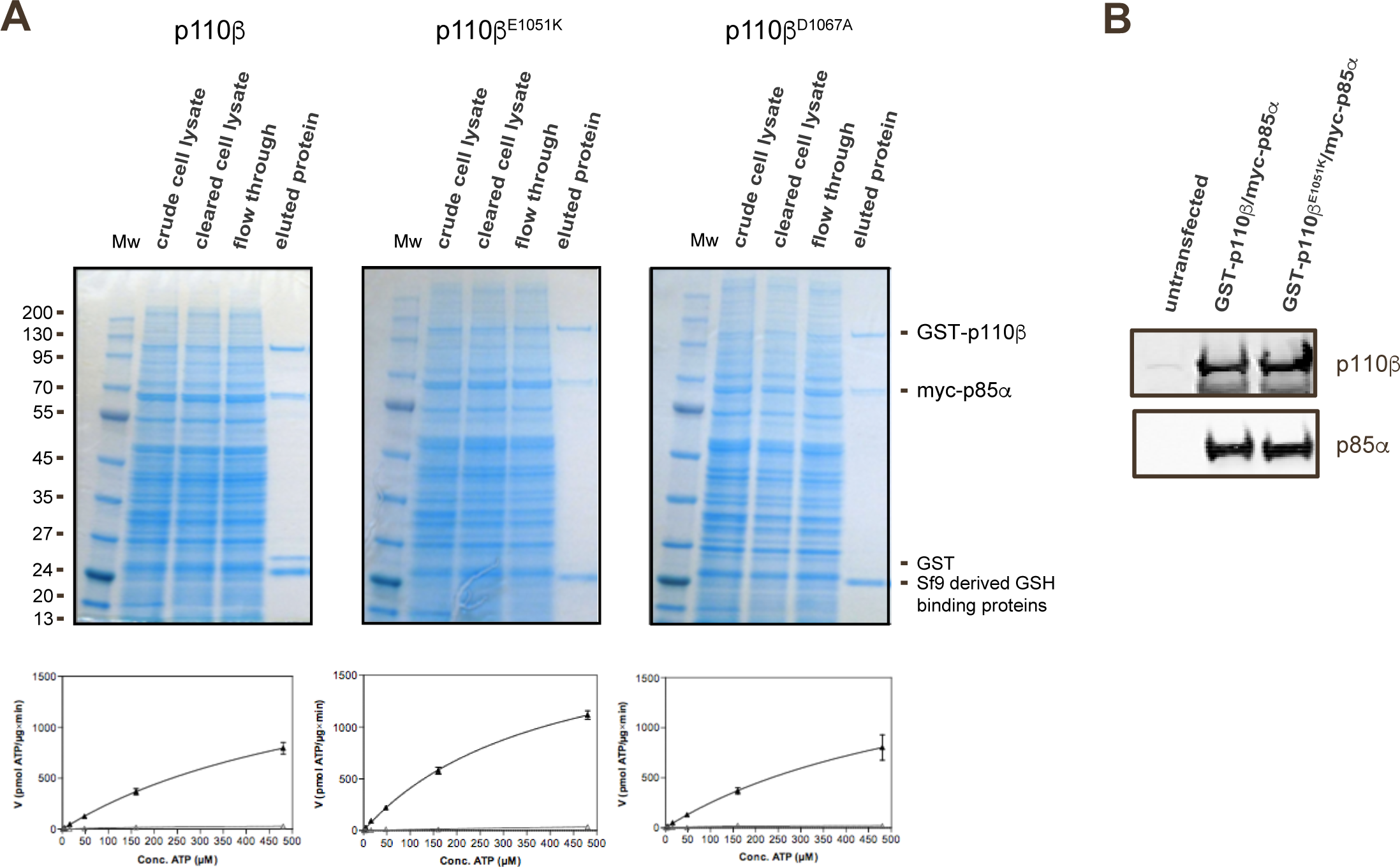

Supplement: Supplementary Figure 2 [file sigtrans201763-s2.tiff]
