# Supplementary material for: Functional characterization of a novel somatic oncogenic mutation of PIK3CB
Source: Signal Transduct Target Ther. 2017 Dec 22;2:17063–. doi: 10.1038/sigtrans.2017.63 (PMC5740215; doi:10.1038/sigtrans.2017.63)
Supplement: Supplementary Figure Legends [file sigtrans201763-s3.doc]

**Supplementary Figure 1.** (A) Coomassie stained SDS-PAGE gels. Sf9 cells expressing p110β-p85α were lysed, and cellular debris and the insoluble fraction were removed by centrifugation. The cleared lysate was applied to a glutathione (GSH)-affinity matrix, GST-tagged protein was bound, and the beads were washed before elution by free reduced GSH. Samples taken at each stage of the process were analyzed by SDS-PAGE, and proteins were visualized by Coomassie stain. One major contamination band could be observed at an approximate molecular weight of 24 kDa, consisting of insect cell derived GSH-binding protein. A gel from a preparation of p110β^D1067A^-p85α (a distinct mutation that does not impair kinase activity) is included for comparison. Plots below the gels depict lipid kinase assays performed on samples of each purified mutant p110β-p85α preparation tested for *in vitro* lipid kinase activity using the assay conditions described in Materials and Methods. (B) Immunoblot of Sf9 cell lysate or Sf9 lysates of cells expressing GST-p110β (wild-type or E1051K)/myc-p85α were probed using antibodies to detect expression of p110β and p85α.

**Supplementary Figure 2.** p110β** **protein expression. Immunoblot of whole cell lysates of indicated cell lines cultured overnight in media containing 10% fetal bovine serum probed using antibodies to detect the level of class IA PI3K p110 isoform and GAPDH expression. A total of 20 μg of protein was loaded in each lane. Fluorescent intensity of the p110β band was quantified and normalized to GAPDH. For comparison, the p110β signal normalized(normalized to GAPDH) derived from the blot in Fig. 2A from parental, mock and myc-p110β and myc-p110β^E1051K^ transduced Rat2 cells is shown.
